# Developing Next-generation Brain Sensing Technologies – A Review

**DOI:** 10.1109/jsen.2019.2931159

**Published:** 2019

**Authors:** Jacob T. Robinson, Eric Pohlmeyer, Malte C. Gather, Caleb Kemere, John E. Kitching, George G. Malliaras, Adam Marblestone, Kenneth L. Shepard, Thomas Stieglitz, Chong Xie

**Affiliations:** 1. Department of Electrical and Computer Engineering, Rice University, Houston, TX 77005, USA.; 2. Department of Bioengineering, Rice University, Houston, TX 77005, USA.; 3. Department of Neuroscience, Baylor College of Medicine, Houston, TX 77030, USA.; 4. John Hopkins University Applied Physics Laboratory, Laurel, MD 20723, USA.; 5. SUPA, School of Physics & Astronomy, University of St Andrews, St Andrews KY16 9SS Scotland, UK.; 6. Time and Frequency Division, NIST, 325 Broadway, Boulder, Colorado 80305, USA; 7. Electrical Engineering Division, Department of Engineering, University of Cambridge, Cambridge CB3 0FA, UK.; 8. MIT Media Lab, Massachusetts Institute of Technology (MIT), Cambridge, MA 02139, USA.; 9. Department of Electrical Engineering, Columbia University, New York, NY 10027, USA.; 10. Institute of Microsystem Technology, Laboratory for Biomedical Microtechnology, D-79110 Freiburg, Germany.; 11. Cluster of Excellence BrainLinks-BrainTools, University of Freiburg, 79110 Freiburg, Germany; 12. Bernstein Center Freiburg, University of Freiburg, 79104 Freiburg, Germany.; 13. Department of Biomedical Engineering, The University of Texas at Austin, Austin, TX 78712, USA.

**Keywords:** Neural engineering, Sensors

## Abstract

Advances in sensing technology raise the possibility of creating neural interfaces that can more effectively restore or repair neural function and reveal fundamental properties of neural information processing. To realize the potential of these bioelectronic devices, it is necessary to understand the capabilities of emerging technologies and identify the best strategies to translate these technologies into products and therapies that will improve the lives of patients with neurological and other disorders. Here we discuss emerging technologies for sensing brain activity, anticipated challenges for translation, and perspectives for how to best transition these technologies from academic research labs to useful products for neuroscience researchers and human patients.

## I. Introduction

New sensing technologies capable of recording from large numbers of neurons for extended periods of time could fundamentally improve our understanding of the brain and the treatment of neurological disorders. Recent advances in sensing technologies from a variety of research domains promise to provide just such improvements in neural interface technology, but each of these approaches have unique technological challenges and opportunities.

As part of the IEEE Brain Initiative, we recently gathered a group of researchers and stakeholders in Glasgow, Scotland to discuss the current state of the art for some of these emerging sensing technologies and the opportunities and challenges they present. This review focuses on emerging brain sensing technologies that currently reside in the academic research domain and may come to market in the coming years. For readers interested in brain sensing technologies that are currently commercially available, we encourage them to explore one of the excellent recent reviews of commercial brain sensing technologies [1]–[3].

In preparing this review we identified three areas where advanced sensors could disrupt brain sensing technology: 1) New electrode technologies (Section III), 2) Integrated optical sensors (Section IV), and 3) Magnetic field sensors (Section V). In Section VI we summarize the panel discussion regarding the opportunities and challenges for emerging sensing technologies. Before exploring each of these technologies in detail, we first describe the sensing challenge facing brain interfaces and potential opportunities for these sensing modalities. After describing recent efforts in these areas, we conclude with a summary of a panel discussion that occurred during the Glasgow meeting where we discussed grand challenges for the brain sensing community.

## II. Challenges for brain sensing and opportunities for new techniques

**Fig. 1 |:**
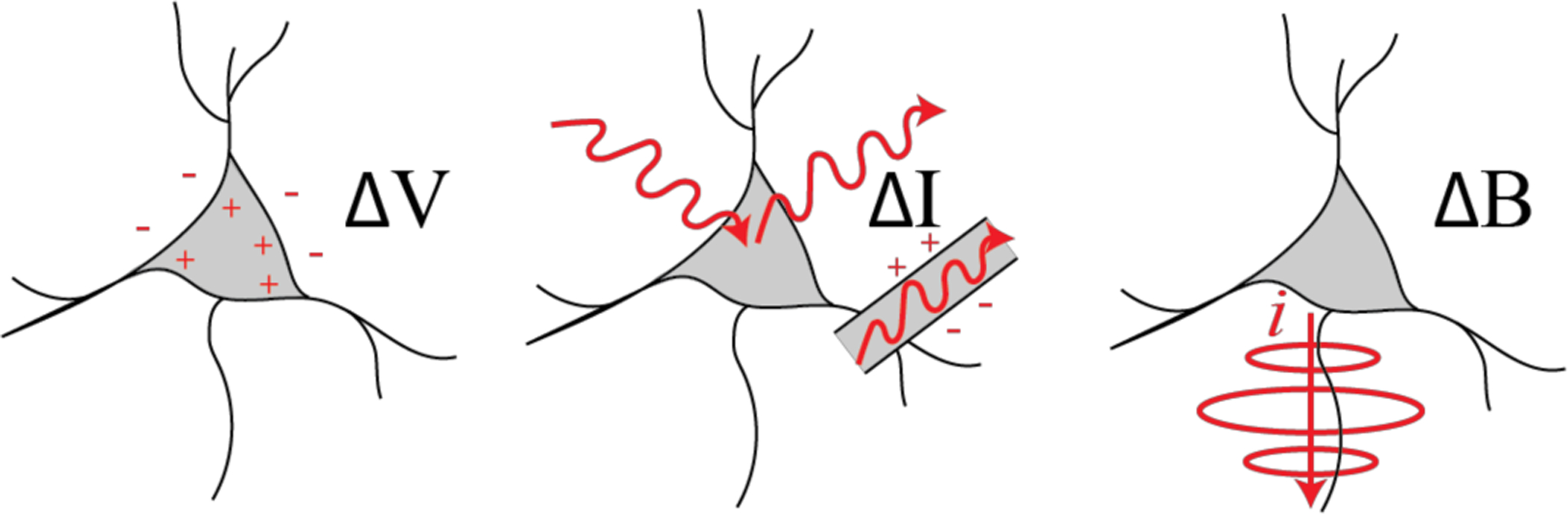
Sensing modalities for measuring neural activity discussed in this review. (Left) Neural action potentials can measured based on changes in electrical potential near the cell body, Section III. (Center) Action potentials can also be detected with light based on changes in fluorescence or changes in transmission of electro-optic waveguides, Section IV. (Right) Neural activity can also be detected based on the magnetic fields produced by currents propagating along neural processes, Section V.

At a fundamental level, sensors that detect the spiking activity of individual neurons convert sub-millivolt voltages within the brain into electronic signals in the solid-state circuits where all data processing occurs [4] (Fig. 1). This conversion is typically performed using an electrical interface between the electrolyte solution in the brain and a metal or organic electrode and associated electronics [5]. Alternatively, light can act as an intermediary where electrical activity is encoded into the intensity of light that is scattered, absorbed [6], for emitted through fluorescence [7], [8] or bioluminescence [9], [10]. It may even be possible to sense this electrical activity by modulating light that passes through a waveguide with electro-optic elements [11]. The currents produced during spiking activity also produce magnetic fields that can be detected noninvasively outside the brain [12].

Each of these sensing modalities have unique opportunities and challenges that we address in turn in the sections below.

## III. Electrical sensing of brain activity

Electrodes have the advantage of direct transduction of the voltage produced by neurons, but require close proximity to the cells they intend to study, placing a premium of technologies that minimize damage to the brain. In neuroscience, these electrodes can record voltages in the surrounding electrolyte; stimulate voltages in surrounding electrolyte; patch neurons [13], measuring voltages and currents; or perform electrochemical analysis of redox-active compounds, such as neurotransmitters [14]–[18]. Each of these applications puts different requirements on the electrodes (and the interfacing electronics). In other words, electrodes need to be designed together with the electronics to which they are connected.

Direct electrical transduction brings many advantages, but in contrast to the remote sensing possible with optical techniques, electrodes must be within roughly 100–200 microns of an electrically active cell to isolate action potentials. This requirement for close proximity raises challenges in creating electrodes with appropriate form factors. Biological systems are curved and malleable, while solid-state devices are hard and flat, a difference that can be handled in one of two ways. One can miniaturize the device with respect to the biological tissue (e.g. ultra-small solid-state devices), or one can create solid-state devices that can conform to biological tissue (e.g. flexible or pliable electronics) [19].

### A. CMOS bioelectronics for brain sensing

When choosing to employ electronic interfaces, there are enormous advantages to integrating these interfaces with state-of-the-art complementary metal-oxide-semiconductor (CMOS) electronics. First, integration of electronics close to the electrodes improves signal fidelity by delivering gain closer to the transducer, improving noise performance. CMOS-integrated electronics also enable dense electronics, having the potential to mirror the kind of densities achieved in CMOS imagers. Time-multiplexing of amplifiers is another technique used to optimally exploit the density of electronics available [20].

Electrodes themselves fall into two main categories: Faradaic and non-Faradaic [21]. Non-Faradaic electrodes are sometimes also called ideal polarized electrodes (IPE); they present a capacitive interface with no dc current conduction. Electrophysiology electrodes are essentially non-Faradaic. Faradaic electrodes, in contrast, allow dc current flow and are needed if electrochemical analysis is performed. For non-Faradaic electrodes, electrode impedance is usually measured at 1 kHz and it is essentially the magnitude of the capacitive impedance of the electrode, or |1/wC|. Optimizing non-Faradaic electrodes typically is synonymous with increasing the capacitance of the electrode, which is usually done by increasing the effective capacitance per unit area. Increase in capacitance is usually accomplished by increasing the surface area through a “porous” surface, such as platinum black and TiN. Another approach is the addition of organic conducting polymers such as PEDOT:PSS [22] as described below. Scaling the electrodes to smaller capacitance values increases the noise on the electrode according to kT/C, although more practical limitations on scaling the capacitance on the electrode may come from the interfacing electronics and the associated capacitance divider, which reduces signal.

Active CMOS multielectrode arrays, employed primarily for in vitro neural studies such as of the retina, set the standard for the density and scaling of the electronics for electrophysiology. Recent work has produced arrays supporting as many as 65k simultaneous recording and stimulating channels [23]. To expand these efforts to in vivo measurement, one of four form factors is required:

#### Penetrating Shanks (flexible and rigid).

Both passive [24] and active [25] versions penetrate the cortex and transmit data wireless or via wires to the surface. Form factors include single or multi-electrode and some flexible versions can be injected through syringes or using fluidic microdrives as described below.

#### Surface recording arrays.

These are both passive [26] and active [19] and are capable of recording both local field potentials (LFPs) and single-unit responses (action potentials). These can be both wired and wireless.

#### Ultra-small free-floating motes.

These must generally communicate wirelessly to the surface of the brain or skull.

There have been several efforts associated with the development of passive silicon shanks. By “passive,” we mean that the shanks themselves have no active electronics. With advanced packaging approaches and custom application-specific integrated circuits (ASICs) for the electronics, passive silicon shanks have been scaled to 1024 electrodes on a single shank. Active CMOS shanks integrate the electronics on the shank itself and can achieve similar scales. Ultra-small passive wired electrodes can be injected [27] or developed into large bundles [28]. There are also passive and active surface recording electrodes using flexible polymer materials as substrates. Active CMOS arrays can also be created by thinning silicon CMOS down to thicknesses below 15 mm, rendering these devices flexible and pliable [29]. Ultra-small wireless implants generally require other energy modes for communication and telemetry at depth, including ultrasound [30]–[32], or magnetic fields [33].

Electrical interfaces continue to be an important type of neural interface and maintain several advantages over optical approaches, however they are generally more invasive and require close proximity with the target neurons. The optimal choice of electrode type depends on the problem at hand, and it is often critical to codesign the electrodes and the associated electronics. Scaled CMOS electronics is an important part of the story, and CMOS can be shaped into unusual form factors. Flexibility and small sizes are keys to building these interfaces.

Important research topics in electrode development include organic electronics that can increase the capacitance of the electrode-electrolyte interface, nanoscale form factors that can reduce a foreign body response from the tissue, and minimally invasive delivery strategies that can improve chronic electrode performance. These research areas are described in the following sections.

#### B. Organic bioelectronics for brain sensing

Organic synthesis allows one to create bioelectronic materials with tunable properties, compatibility with flexible substrates, and mixed electronic/ionic conduction [34], [35], all of which have advantages for brain sensing and can be combined with CMOS bioelectronics. This mixed electronic/ionic conductivity, in particular, enables one to lower the impedance of neural electrodes. For example, a thin (~100 nm) coating of a commercially available conducting polymer such as PEDOT:PSS, can lower the impedance of a Au electrode by a factor of 100 [36]. When such electrodes are integrated with thin plastic films, they allow the fabrication of ultra-conformable cortical grids that provide exceptional spatial resolution. One example is the NeuroGrid, a parylene-based conducting polymer microelectrode array that allows single neuron recordings without penetrating the cortex [37].

The same property of mixed conductivity allows the fabrication of electrochemical transistors. In these devices, ions from the cerebrospinal fluid enter the polymer channel and change the electrical conductivity throughout its volume [38]. This mechanism of operation is associated with a very large transconductance and these transistors act as amplifying transducers, recording neural activity with higher signal-to-noise ratio than electrodes of the same size [39].

Moreover, electrochemical transistors can be functionalized with redox enzymes, acting as sensitive sensors for metabolites such as glucose and lactate. Recent work shows that these devices offer good stability in cell culture media and that they can measure changes in metabolic activity with a high sensitivity [40]. These devices are now *en route* to implantable applications, with the aim of combined measurements of electrophysiology and metabolism in the same location of the brain. In the form of organic light-emitting diodes (OLEDs), organic electronics can be a useful way for local generation of light as will be described in Section IV, B.

#### C. Nanoelectronics for improving long-term neural interfaces

As described above, electrodes are one of the most important effective methods for time-resolved electrical detection of individual neuron activities in the living brain[41]–[47]. However, reliable tracking of the same groups of neurons over days to months and years with neural electrodes has been challenging, mainly due to their unstable performance over long-term implantation [48]–[50]. The stresses in the implants induced by the motion of the surrounding tissue often leads to structural damages of the neural electrodes and abrupt loss of recording capacity [51]–[54]. Moreover, these implanted electrodes also induce substantial damage to the host tissue [55]. This damage is often attributed to the large dimensions, surgical footprints, and mechanical rigidity of stiff neural electrodes. In the short term, the mechanical mismatch between tissue and implants induces electrode movements with respect to target neurons [56], which leads to sudden waveform changes on time scales as short as hours, preventing reliable tracking of individual neurons over days and longer [57], [58]. In the long term, the presence of implants causes recurring cellular and vascular damage, elicits sustained inflammation and tissue response [59] that results in neuronal degeneration and glial scar formation near the implants [55], [60], [61]. These chronic deteriorations are manifested in electrical recordings as loss in recording yield, amplitude and fidelity [48][61]–[64], greatly limiting their application in both fundamental and clinical neuroscience.

There has been a growing awareness that reducing the neural probe’s dimension [66] and rigidity[61], [67], [68] can improve the tissue-probe interface, raising the prospect of improved chronic performance for tiny flexible electrodes. Recent demonstrations of ultra-flexible nanoelectronic threads (NETs) [65] and flexible electrode meshes [69] indeed show improved stability of neural recording. For example, NET electrodes can reliably detect and track individual neurons for over three months; and the electrode impedance, noise level, single unit yield, and the signal amplitude remained stable over long-term implantation. In vivo two-photon imaging and postmortem histological analysis show fully recovered capillaries with an intact blood-brain barrier, absence of chronic neuronal degeneration and glial scar. Future work is underway to scale up these ultra-flexible electrodes to achieve large-scale, high-density and long-term neural recording [70], and combining electrical measurements with optical imaging [71], [72].

#### D. Realizing the promise of flexible electrodes via microfluidic insertion

While very small and/or very flexible electrodes are likely to improve chronic recording performance [73], [74], devices that are small and/or flexible are typically quite fragile, and thus difficult to insert into the brain. As a result, most solutions which accommodate scales of 100s or 1000s of electrode sites currently use some form of transient stiffening to insert devices into the brain. The two most common forms of transient stiffening are dissolvable coatings and the attachment of a stiffener with a dissolvable adhesive or temporary mechanical connection [75]–[77]. The problem with these solutions is that the stiffened devices still cause trauma during insertion, in particular, damaging the microvasculature of the brain in the areas in which they are inserted [78], [79].

**Fig. 4 |:**
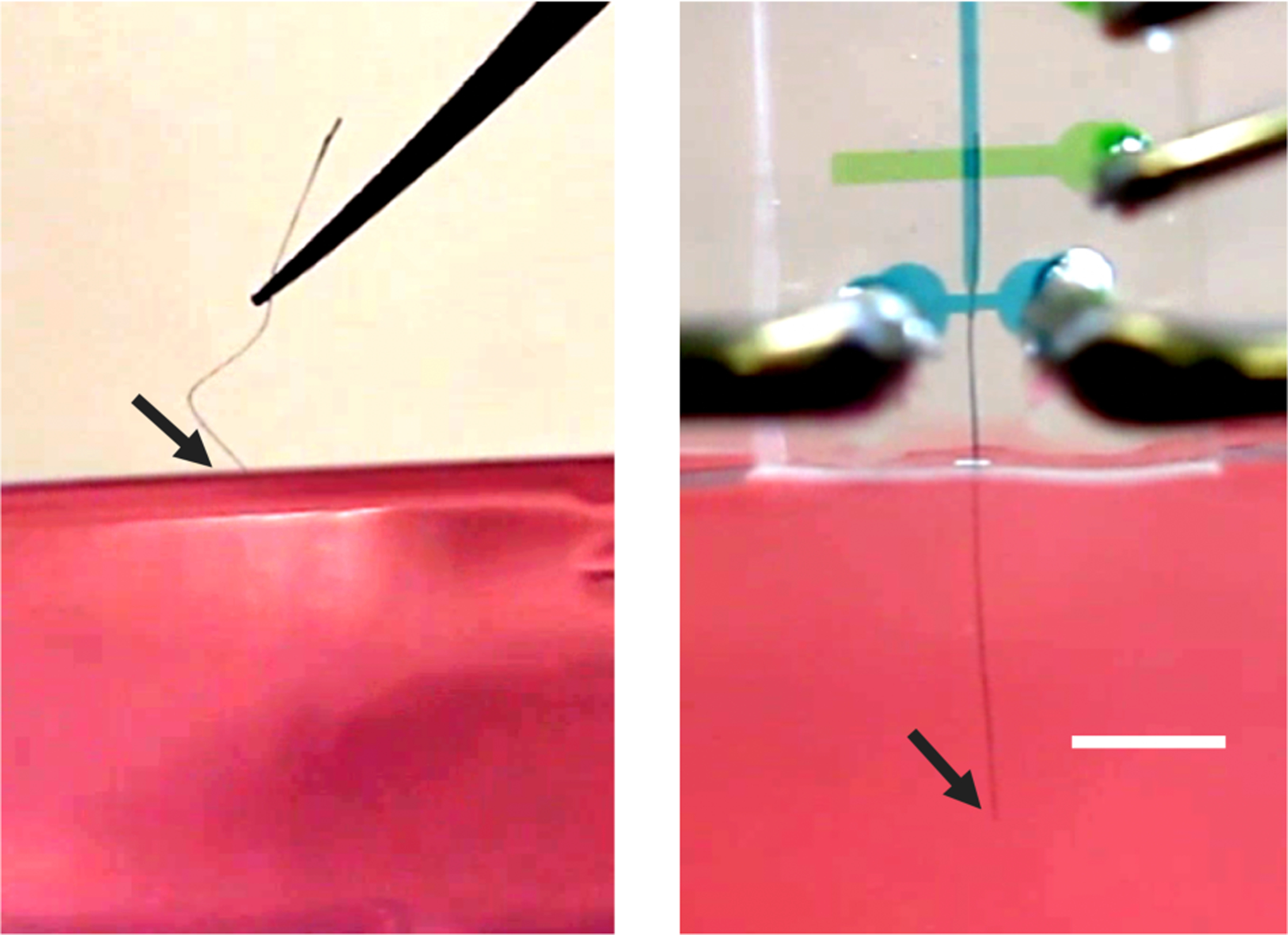
Ultraflexbile carbon nanotube fiber (CNTf) probes buckle when pressed into an agar brain phantom (left). A fluidic microdrive that supports the bare fiber during actuation facilitates implantation without increasing the footprint of the electrode (right) [80]. Scale bar 2 mm.

In order to minimize insertion trauma, researchers have developed a novel technique to insert flexible probes into the brain without stiffening [80]. For any probe, the primary mechanism of insertion failure is buckling in regions that are unsupported, i.e., between the brain surface and the location at which force is applied [81], [82]. Using microfluidic channels to support the electrode along the entire length it is possible to increase the amount of force the probe can tolerate before buckling as it is inserted. Within the microfluidic channel, drag from high speed fluid flow drives the electrode into the brain. Large return ports proximal to the end of the channel capture 98.5% of the fluid to ensure that the drive fluid does not penetrate or damage the brain. Thus, while this approach shares some similarity with a recently-reported syringe injection-based approach [27], [83], neither the drive channel nor the fluid are able to damage the brain during electrode insertion. Using this fluidic microdrive, researchers have successfully inserted single channel flexible carbon nanotube fiber electrodes into model organisms (the cnidarian Hydra vulgaris), acute mouse brain sections, and the in vivo rat brain. With this approach it is also possible to control the depth of the probe by actuating the drive fluid pressure [80]. Subsequent work has also demonstrated that using a dissolvable polymer support can also stabilize a flexible implant near the insertion point to help prevent buckling [82] (Fig. 4). By removing the need for a stiffener, it may be possible to simplify the process of electrode insertion by removing the need for dissolution of an adhesive as well as significantly reducing the damage done to the brain during implantation. Improved methods like these for implanting small and highly flexible electrodes will be instrumental in facilitating the next generation of high-channel count chronic neural electrode arrays.

#### E. Stability and functionality of flexible electrodes

In addition to improving system integration and performance of the electrodes at tissue interface, long-term studies in fundamental neuroscience as well as clinical applications in humans also require that these interfaces are robust and reliable with life times from years to decades. While there is plenty of knowledge in the medical devices field with cardiac pacemakers, cochlear implants and deep brain stimulators, the underlying technologies using precision mechanics limit the degree of miniaturization and the number of electrodes and cannot be directly transferred into miniaturized neural implants with dozens to hundreds of channels [84].

As described above, CMOS and other micro (opto-) electromechanical systems (M(O)EMS) offer adequate levels of miniaturization but still have to prove long-term stability in chronic preclinical and clinical settings [84]. Package, substrate and electrode materials have to be stable in the body, must not be toxic and as described above should minimize the mechanical mismatch between the neuronal target tissue and the implant to limit foreign body reaction due to structural biocompatibility issues.

One approach for neural interfaces applying MEMS technologies uses polyimide as flexible substrate and insulation material and thin-film metallization for electrodes, interconnect lines and contact pads to cables and implant packages. Adhesion between the different materials and layers has been identified as one of the key properties for long-term stability [84]. Reliable assembling to either telemetric systems or wired connectors is mandatory for recording of nerve signals or electrical stimulation. Devices have been designed according to the anatomical and neurophysiological targets in the central and peripheral nervous system. Customized electrode arrays for chronic recording of electrocorticograms have been developed [85] for ferrets [86] and non-human primates [87] with wired connectors. Modular device designs go currently up to 1024 channels without the need of multiplexing electronics. However, the percutaneous headposts limit transition into clinical settings, at least in Europe. Fully implantable, wireless systems [88] overcome this limitation but have to time-multiplex the transmission of data. Signal processing and data compression or algorithms on the implant reduce the amount of data to be transferred. In the peripheral nervous system, mechanical forces on the implant due to muscle contractions have to be considered in system design. Functional interfaces have been miniaturized and combined with robust cables in the TIME concept [89] for interfacing arm nerves after amputation to deliver sensory feedback in hand prosthesis control in subchronic conditions [90] and up to six months [91]. The lack of high-channel count implantable connectors currently limits the usability and translation of these approaches into medical devices for clinical use worldwide. Optoelectronic probes for optogenetic applications face similar challenges [92]. Thermal management of implanted light sources, their hermetic packaging and the transparency of waveguides are current bottlenecks [93] for long-term application.

Foreign body reaction leads to scarring reaction around the implants deteriorating the transfer properties of the electrical and optical channels. Carbon as electrode material delivered promising results with respect to reduced tissue reaction [94]. Incorporation of drugs in conductive polymers and delivery on demand [95] is another option to actively control reactions at the material-tissue interface for long-term functional interfaces to the nervous system and reliable performance of neural implants.

Thus, in addition to carefully developing sensors at the biotic-abiotic interface, it is critical to design systems for data and power transfer that support these sensors over long periods of time in the harsh and delicate environment inside the body.

## IV. Optical brain computer interfaces

Using light to sense neural activity has several advantages compared to electrical interfaces. Photons in brain tissue typically travel between 50 and 100 microns before scattering, allowing one to image individual cortical neurons from the surface of the brain. This imaging ability combined with advances in designer proteins for stimulation and measurement, allows one to interface with select cell types [96]–[98]. This cell-type-specific information combined with less invasive interrogation, and the ability to image many cells at once is raising interest in optical brain interfaces.

Light, however, is not without disadvantages. Information transferred through photons is limited by shot noise, which is most pronounced at high bandwidths and at the low signal levels characteristic of optical reporters of neural activity. The use of light is also an indirect measure of biophysical processes since, in nearly all cases, biological systems must be engineered to emit or be sensitive to light. Photobleaching also limits measurement time. Instrumentation is complex – microscopes are big and bulky generally, despite recent advances in lensless and filterless imaging systems. To overcome some of these challenges, researchers are investigating ways to make miniature, lensless microscopes with dramatically reduced size and weight, and looking toward electrooptic effects that would eliminate the challenges associated with photobleaching.

### A. Flat lensless microscopes for implantable optical interfaces

Chief among the challenges facing an optical brain computer interface is the creation of a fully implantable imaging system that covers significant areas of the cortex. While miniature microscopes have shown promise for imaging activity in freely moving animals [99], [100], these microscopes rely on traditional architectures that use lenses to magnify the image. As a result, the imaging system must be much larger than the field of view, making it difficult to image large areas while maintaining the small form factor necessary for an implanted device [99]. Thus, small microscopes traditionally have small fields of view.

**Fig. 5 |:**
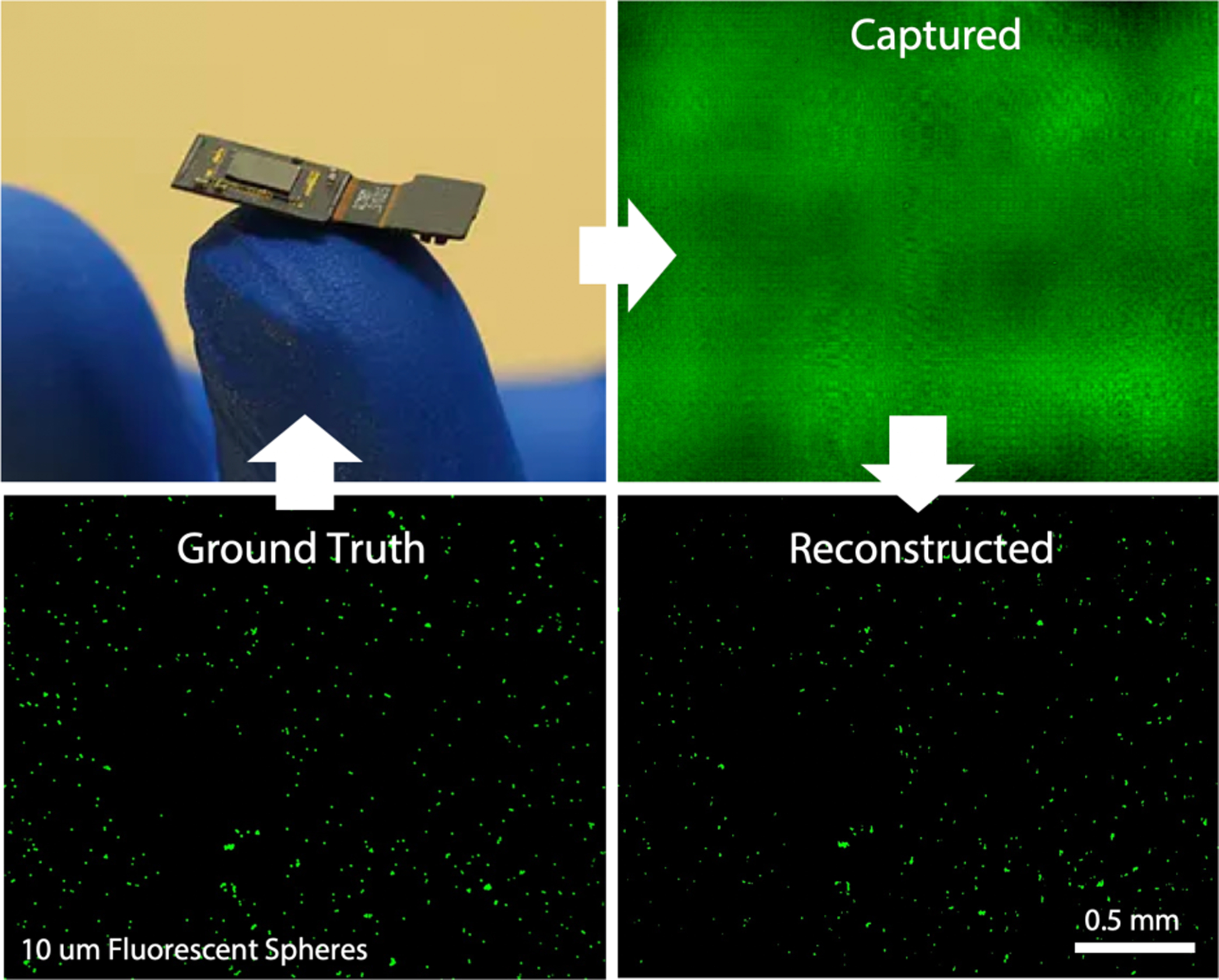
FlatScopes like the one shown on the tip of a figure have no lenses making them ultrathin and lightweight. However, without lenses, the captured images do not resemble the ground truth (shown here in a confocal microscope image of fluorescent microspheres). Nevertheless, by using specially designed diffractive or amplitude masks placed directly on the sensor, one can computationally reconstruct an image that closely matches the ground truth [101], [102].

One approach to overcome the limitations inherent to lens-based microscopes is to replace lenses with compact phase or amplitude masks and computational imaging algorithms to recover an accurate estimate of the scene based on complex sensor data (that may not initially look like an image). One approach called “FlatScope” is a device less than a millimeter thick that maintains micron-scale resolution over a field of view several millimeters across (Fig. 5). In addition to the compact form factor, computational imaging employed by FlatScope allows one to refocus images to different depths, which enables 3D volumes to be reconstructed from a single image capture. Thus, high-frame-rate 3D images can be reconstructed to potentially reveal neural activity across neural circuits [101].

While flat, lensless microscopes may solve the size and weight challenge for optical interfaces, there remains a need for brighter and more stable fluorescent indicators of neural activity, as well as more sensitive image sensors that can be incorporated into devices like the FlatScope. Promising new single-photon-sensitive sensors may provide just such advances in imaging technologies. Secondly, to image beyond the first few hundred microns of cortex requires technologies to mitigate the effects of light scattering by brain tissue. While multi-photon microscopy is the most common method to increase the optical imaging depth, it is unclear if the required pulsed lasers can be integrated into chip-scale neural interfaces. One alternative could be to produce light within the brain via bioluminescence to overcome the effects of excitation scattering and enable deeper imaging. Alternatively, integrated photonic probes that produce sheets of excitation light could more than double the imaging depth achieved by epifluorescence microscopy [103].

### B. Organic LEDs for brain optical stimulation and imaging

While advances in CMOS technology allow production of fast and sensitive megapixel image sensors, generation of light by miniature integrated electronics remains a challenge, limiting its use for all optical neuronal interfacing, which requires photostimulation of neurons, e.g. through optogenetics, and in many cases excitation of fluorescent voltage or calcium reporters. This is a fundamental limitation of silicon due to its indirect bandgap. One avenue to circumvent this issue is to bond conventional micro LEDs, e.g. based on GaN, onto the silicon chip [104]–[106]. However, due to the lattice mismatch of GaN and Si, this integration requires involved post-processing, e.g. through flip-chip bonding, which to our knowledge has so far prevented development of megapixel active matrix CMOS LED arrays for optical neuronal recording or optogenetic stimulation.

In recent years, researchers have begun to use monolithic integration of organic-semiconductor based LEDs (OLEDs) on CMOS chips. Due to their amorphous nature, organic-semiconductors can be deposited on a wide variety of substrates using conventional vacuum techniques like physical vapor deposition or even solution-based approaches. This characteristic of the OLED technology is the principal reason for its great success in the display industry, from small smartphone displays to large TVs. Further benefits of OLED technology in the context of brain-computer interfaces are their intrinsic mechanical flexibility that allows integration on flexible and thus potentially less invasive devices, the ease of color-tuning across the entire visible range of the spectrum, and the fact that their physical dimensions are readily scalable from many cm^2^ down to the μm^2^ range. By modifying OLED microdisplays that were originally developed for near-to-eye display applications, we demonstrated photostimulation of channelrhodopsin 2 (ChR2) expressing cells from >10^5^ individually addressable blue-emitting OLED pixels [107], [108]. A potential weakness of OLEDs is their sensitivity to water and oxygen, and so we have optimized thin-film encapsulation and passivation methods based on atomic layer deposition to enable prolonged operation of OLEDs in a tissue environment [109]. Using the concept of molecular doping, we have also realized OLEDs that achieve brightness levels 100–1000 fold higher than conventional displays and have demonstrated that these allow robust photo-stimulation of individual neurons and small animals genetically transduced to express the latest generation channelrhodospins as well as genetically encoded calcium indicators [110], [111].

### C. Electro-optics for ultraminiature brain sensors

Light may also provide an answer for ultraminiature multichannel brain sensors. While the multi-site electrical neural probes describe above have enabled multiplexed neural activity measurements [25], their level of miniaturization is constrained by several factors. First, in current implementations, each recording site uses a dedicated electrical trace (i.e., a wire, often lithographically integrated into a substrate) to convey its signal to the outside world or to a relay station where amplification and/or digitization may occur. After the digitization stage, many neural signals may be transmitted along a single electrical wire, but prior to this stage, multiplexing requires one wire per recording site. As wires become thinner, we run into limits of fabrication as well as of increasing electrical Johnson noise with increasing electrical resistance [11]. Moreover, if we wish to record at high density at many sites along a long shank, e.g., centimeters in length, the number of wires which must be packed into a single probe increases, or alternatively, power-consumptive amplification and digitization stages must be placed closer to the measured neurons, increasing the complexity of heat dissipation and introducing additional safety considerations.

One potential solution to these problems is to multiplex neural signals optically, into an optical fiber. Because visible or infrared light contains terahertz (THz) frequencies, the available signal bandwidth in an optical context is very high, concordant with the use of optical communications for long-range data transfer in the telecommunications industry and increasingly in parallel computing hardware. Optical communication channels, in addition, can potentially shrink towards the size of the optical wavelength, e.g., in the range of one micron, or below using plasmonic or other optical confinement techniques. Because the light is confined to an optical fiber in such a scenario, the massive optical scattering of the brain tissue does not pose a problem.

To implement such optical multiplexing, there are two possibilities. One is to amplify and/or digitize locally and then transmit via optical fiber. This has some of the same issues, however, in terms of size and power consumption as a pure electrical approach. An alternative possibility is to leverage electro-optic modulation to allow an un-amplified neural signal to be directly transduced into a detectable modulation of an optical wave traveling in a small optical fiber. Electro-optic modulator devices which can create such effects are, interestingly, now scaled down to the micrometer size scale [112], and moreover, although they typically use 1V-scale electrical voltages and switch in the gigahertz (GHz) range, both the speed (kHz) and size of signal (100 microvolt), are proportionately reduced in a neural sensing application. In addition, time-domain or frequency-domain reflectometry techniques can now reach ~10 micrometer spatial resolution within optical fibers [113], and low-coherence reflectometry in optical fibers can achieve similar high resolution and high speed performance as free-space optical coherence tomography which is often applied on the micron scale as well [114].

Recently, preliminary designs have been proposed to allow fiber-based electro-optic neural activity sensing [115]. The design is similar to that of free carrier effect based electro-optic modulators. Based on an analysis of photon shot noise in the system, the authors concluded that neural sensing was possible, if a sufficiently high capacitance can be fabricated inside the proposed engineered waveguide. Improved designs using resonant enhancement of the electro-optic effects via optical cavities, or improved capacitor materials, could allow the still-hypothetical system to operate at lower light powers, reducing heat dissipation from the fiber. With such improvements, as well as improvements in the reflectometry or multiplexing scheme, e.g., wavelength multiplexing, it may be possible to engineer ultra-long (e.g., centimeters), ultra-multiplexed, low-power neural activity sensors for deployment into the brain tissue or via the cerebral vasculature [115]. Achieving this will require creative designs from nano-photonics experts via a cross-disciplinary collaboration with neurophysiologists, materials scientists and experts on optical detection methods.

## V. Magnetoencephalography with atomic magnetometers

Compared of electrical and optical techniques, magnetic sensing of neural activity has the advantage of being completely non-invasive and has high temporal resolution. However, the fields produced are so small compared to the earth’s magnetic field. As a result, the measurement of such fields has been traditionally carried out by magnetometers based on superconducting quantum interference devices [116], which achieve the required sensitivities, but which require cryogenic cooling and are large, expensive and cumbersome to operate.

Recent improvements in the sensitivity of atomic magnetometers [117], which are based on the precession of the spins of alkali atoms in the vapor phase, now allow these sensors to be used for magnetoencephalography [118] and there is growing interest within the biomagnetics community in these sensors [119]. The key advance that led to the improved sensitivity is the reduction of spin-exchange relaxation [120], which can severely limit the sensitivity at high alkali densities. Magnetometer sensitivities at or below 1 fT/√Hz have been demonstrated in the laboratory and commercial magnetometers with a sensitivity of 15 fT/√Hz are available commercially [121].

**Fig. 6 |:**
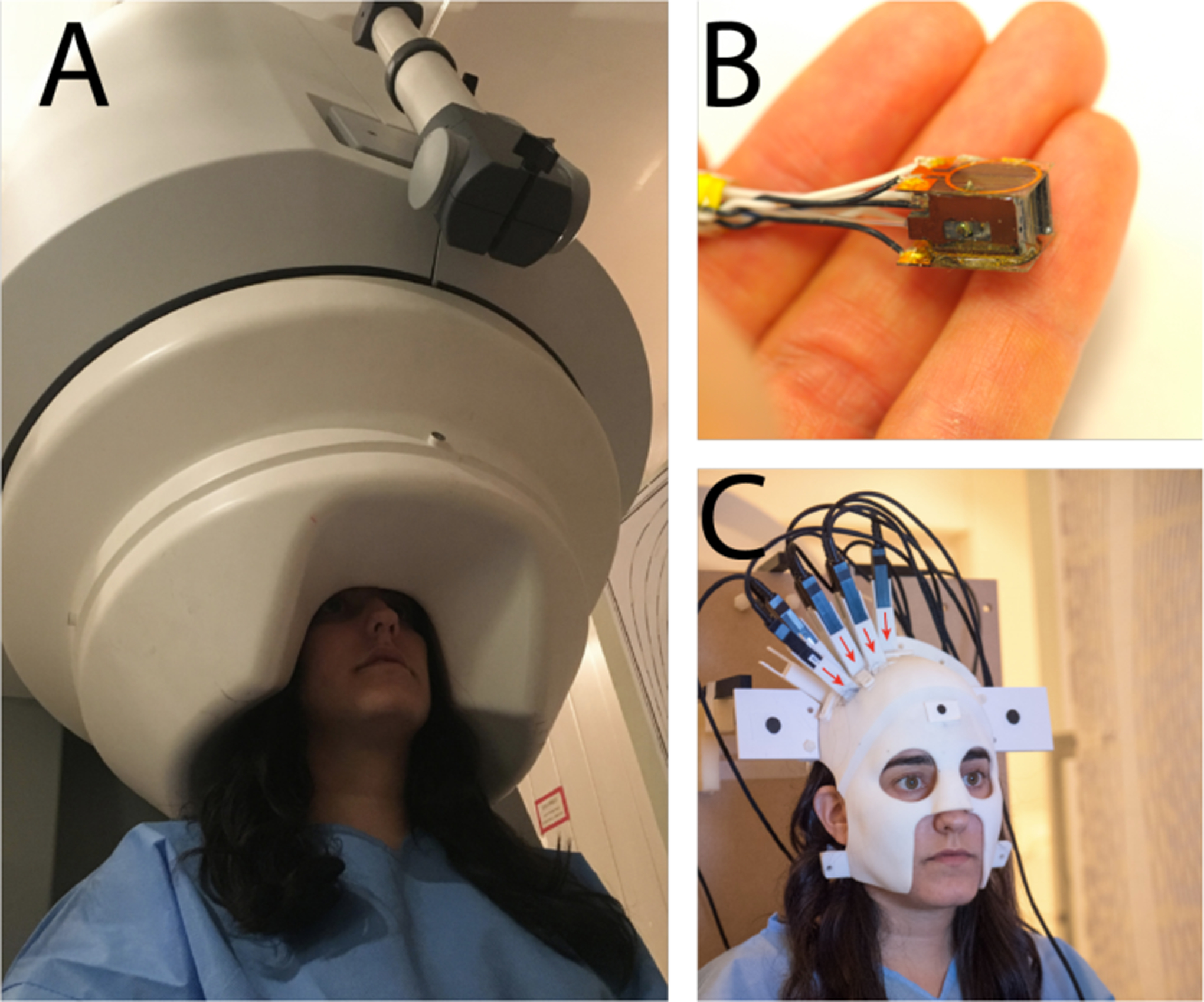
Magnetoencephalography for capturing brain activity. A) Liquid-helium cooled magnetometers for traditional MEG are large and heavy instruments. B) Optical magnetometers, on the other hand, operate at room temperature and can be miniaturized. C) Using miniature optical magnetometers it is possible to create small form factor MEGs that operate at room temperature.

Along with the improvements in sensitivity obtained over the last decade, there have been parallel improvements in miniaturization and manufacturability. Chip-scale atomic magnetometers [122], [123] combine the high precision of alkali magnetometers with the small size afforded by silicon micromachining processes. By confining the atoms in a millimeter-scale micromachined vessel and probing the atomic spins using light from a low-power laser, substantial reductions in power consumption, volume and cost can be obtained (Fig. 6). For magnetoencephalography, the main advantages of chip-scale technology are that high spatial resolution can be obtained, and the sensors can be placed closer to the skull, resulting in improved signal strength. Spontaneous brain activity and evoked responses have been measured [124] in human subjects with chip-scale atomic magnetometers, and epileptic spiking has been measured in rats [125].

It is anticipated that further development of this sensor technology may lead both to portable systems capable of, for example, measuring brain activity during epileptic seizures, and to operation without the stringent shielding requirements required for superconducting quantum interference device (SQUID)-based systems [126]. These, along with further commercial development of the sensor technology and integration into full brain imaging systems, are some of the main challenges which need to be overcome in the coming years.

## VI. Panel discussions: Perspectives on Neural Sensing

As part of the workshop, a panel of neural sensors researchers discussed some of the challenges facing neural interface technology, and proposed suggestions for how they could be addressed. Some of the main themes that emerged from that discussion included: 1) A proposal that flagship research in neural interfaces should be focused on one or more clinical or commercial needs that involves a significant population of beneficiaries 2) Developing application spaces should draw on clearly articulated basic science questions 3) New sensing techniques are needed that change the risk profile associated with neural sensing and 4) The need for translation-based teams to involve people from many different disciplines, including some not traditionally associated with conventional neural interface research.

### A. Flagship applications to benefit neural interface development

A common theme of the panel’s deliberations was the idea that to best advance brain sensor technology, the community should nucleate around high-priority applications rather than focus on arbitrary technical or scientific metrics. Instead, the technical metrics would be driven by the needs of the target application. With this approach it may be easier to clearly articulate a focused set of engineering design constraints and next step research goals that could be used to advance sensor technology, provide a more direct path to clinical translation, and help motivate research support. The panel felt that selecting flagship application(s) should involve identifying a space in which neural interfaces provided a potential means to benefit a larger number of people relative to smaller niche clinical domains. The panel did not feel that identifying one or more specific application spaces would significantly impede neural interface research, as the overlapping needs of many goal applications meant that advancing neural sensor technology for a given goal application would still benefit neural technology at large.

The panel also expressed that many neural sensing technology questions that involved identifying necessary performance metrics and pressing technical challenges were difficult to answer except in the context of specific applications (e.g. desired electrode stiffness, and whether it may be better to maintain that stiffness chronically or transiently). For example, the panel noted that designing systems capable of monitoring single neurons may be necessary to enable brain-computer interface (BCI) control of dexterous prosthetics hands or for basic neuroscience studies, but local field potential (LFP) activity may be sufficient for other BCI applications (e.g. computer cursor control), and thus it was difficult to prioritize a goal sensor resolution for neural interface research as a whole. However, the panel noted that in the case of BCI, the clinical population of severely paralyzed individuals who would potentially benefit from either approach would be relatively small compared to the clinical need in spaces such as pain management, with opiate addiction becoming a national crisis [127], [128], and Deep Brain Stimulation (DBS), which is currently being used by more than 40,000 people worldwide [129]. The panel felt that selecting a domain such as DBS or electroceutical pain management as a high priority neural interface application space would help the research community identify a reduced set of clearly articulated, high-priority technological and scientific goals. The panel expressed many advantages of focusing on applications: 1) accelerating neural interface research while also helping to motivate research investment, 2) providing a flagship platform to explore regulatory restrictions and health reimbursement obstacles to neural interface clinical translation, which would benefit from a larger potential pool of beneficiaries), 3) helping avoid instances of technological hype outstripping its capabilities since concrete application goals would provide clear metrics against which progress could be measured, 4), providing a means for other neural interface areas, such as BCI, to advance given that shared domain challenges (e.g. development of less invasive recording and stimulating techniques).

### B. The need for basic science to support neural sensor development

While the panel advocated identifying one or more high-priority application spaces, it also emphasized the need to improve our basic scientific understanding of the relevant neural mechanisms associated with: 1) the proposed application, 2) the factors affecting device performance, and 3) the information content of the recorded data. The panel expressed the opinion that when considering potential high priority application domains, the degree to which the fundamental problems associated with the application having been historically well studied and well understood should be a significant selection criterion. Similarly, research efforts going forward would benefit by being able to frame their basic science questions in regards to the identified application(s) in order to more clearly articulate their goals. For example, when asked to assess the importance of novel sensing methods ability to monitor individual neurons, the panel pointed out that basic research would be necessary to determine whether single neuron recording fidelity was important for a given application. More specifically, research would be necessary to better quantify the information content of LFP data relative to single neuron recordings, the temporal stability of LFP information, the stability by which single neurons can be tracked over time for a given method, how well information content scaled with additional sensors for single neuron vs LFP sources, etc., Only with such basic work would it be possible to assess the need for single neuron monitoring for a proposed application. Similarly, when discussing the major impediments to current clinical translation efforts, the panel articulated a number of areas for which basic research would be necessary to overcome translation hurdles, including: safety studies regarding optogenetic techniques, establishing the consistency with which specific neurons could be linked to sensor recordings, studies regarding which neural regions should be targeted for a given application, and investigations into the long and short term impacts of parameters such as electrode stiffness on tissue damage and neural monitoring.

### C. Reducing the risk profiles of neural sensors and stimulators would expand the space of new applications

As part of the its deliberations, the panel returned several times to the idea that a major goal for both neural sensing and stimulation technologies should be to reduce risk profiles associated with deploying and using the technologies. New application spaces, whether commercial or clinical, must consider the risk as well as the benefit to the user, which implies that reducing risk significantly expands the space of possible applications that could be used as high-priority focus areas to support neural interface research. For example, basic research evaluating the impact of electrode motion, both over short and long timescales, may show that flexible electrodes significantly reduce the risk profile for implanted electrodes. Similarly, when challenged to identify the major impediments to translating neural interface technology to clinical practice, the panel highlighted that surgical implantation of a device significantly complicated clinical translation opportunities, and even more so impacted potential commercial applications. With regard to the latter, the panel believed that pursuing noninvasive technologies for neural recording and stimulation would facilitate identification of a high-priority, large population application spaces. The panel pointed out there was significant public interest in neural interfaces, extending even to growing DIY (“do it yourself”) communities that are emerging around neural recording technologies (e.g. the OpenBCI project [130], open source EEG [131] and DIY brain stimulation methodologies [132]–[135]. The panel argued that providing a larger number of reliable and effective noninvasive neural sensing technologies would increase commercial and public interest and thus increase the probability of a successful high-profile commercial application. These developments would in-turn provide greater research opportunities for both invasive and noninvasive neural sensing technologies.

### D. Implementing new neural interfaces requires integration of diverse skill sets

The panel also advocated that research efforts should draw on a wide range of knowledge bases and skill sets to effectively move neural interface technology forward. The panel explicitly noted that bridging the gap between biology and engineers was a key hurdle in developing effective new neural sensing technologies, thus encouraging the formation of multidisciplinary teams. The panel emphasized the need for varied expertise to develop new technologies that can successfully translate into a clinical setting. For example, not only would teams need to be able to effectively integrate clinicians, but they would also need individuals skilled in communication and commercial applications, such as people with venture capital expertise. The panel believed that such skill sets are often overlooked to the detriment of translation efforts. For example, effectively obtaining venture capital investment often includes extensive communication skills and being able to not only clearly describe technical achievements, but also being able to clearly communicate future requirements while delineating scientific and engineering risks. Similarly, currently there is often a cultural clinical aversion against using neural stimulation-based approaches for clinical applications, and good communication skills (often themselves a challenge for research scientists and engineers) will be important to help open up new application spaces. The panel also noted that good communication and leadership skills were necessary (but unfortunately often lacking) for coordinating efforts within a given team. Some of the panelists pointed to personal experiences in which they believed that the necessary types of expertise to support individual components of a project, such as clinical testing and different types of engineering sills, had been present in a team, but the main difficulties had been in bringing the components together effectively. Thus, they advocated for teams to draw on members with expertise (to some degree) that crosses different discipline boundaries to help facilitate team communication and integration of a project’s different scientific and engineering components.

### E. Grand Challenges: Coordination, Barriers to Entry, and Details

The workshop concluded with a discussion among breakout groups to identify the grand challenges for Brain sensing. From these discussions a consensus emerged that there are no clear grand challenges for a technology because there is not a singular goal for neural sensing research. Rather, the group reported that the biggest challenges related to the coordinating the many different engineering pieces that must work together for a functional neural technology and creating robust technologies that can be easily distributed. The groups reported that there is currently a huge barrier to entry for neurotechnology developers if they need to create the end-to-end solution. A move toward modular components in neurotech would allow engineers to focus on developing one element of a system that would could integrate into existing systems. The groups suggested that neurotechnology development platforms similar to what has been done in the software industry could also a empower crowd sourced solutions and communities of DIY’ers to contribute to the neurotech movement. Finally, the groups argued that the largest engineering challenge is not unified, but rather a collection of specific technology challenges ranging from device performance, packaging, biocompatibility, robust operation, connectors, wireless power and data, etc. that together must be solved for “surgeon-proof” technologies. Despite these challenges the panels remained optimistic that these are solvable problems over the next decade.

## Figures and Tables

**Fig. 2 |:**
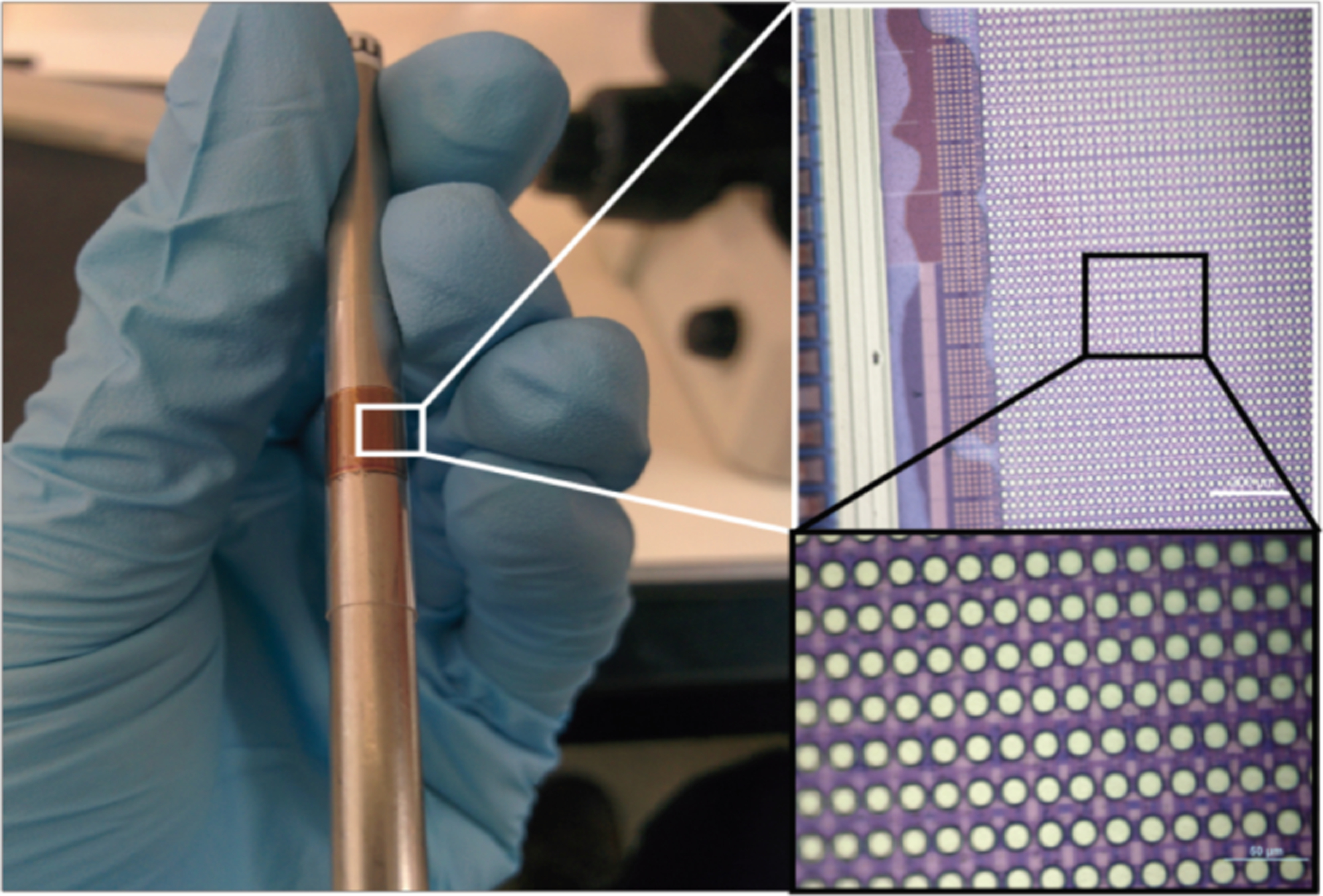
Example of thin, flexible CMOS electronics. When thinned below 15 μm in thickness silicon electronics can bend and flex to match complex topographies like those in the brain to provide close contact with neural tissue. Here we see a flexible array of neural electrodes wrapped

**Fig. 3 |:**
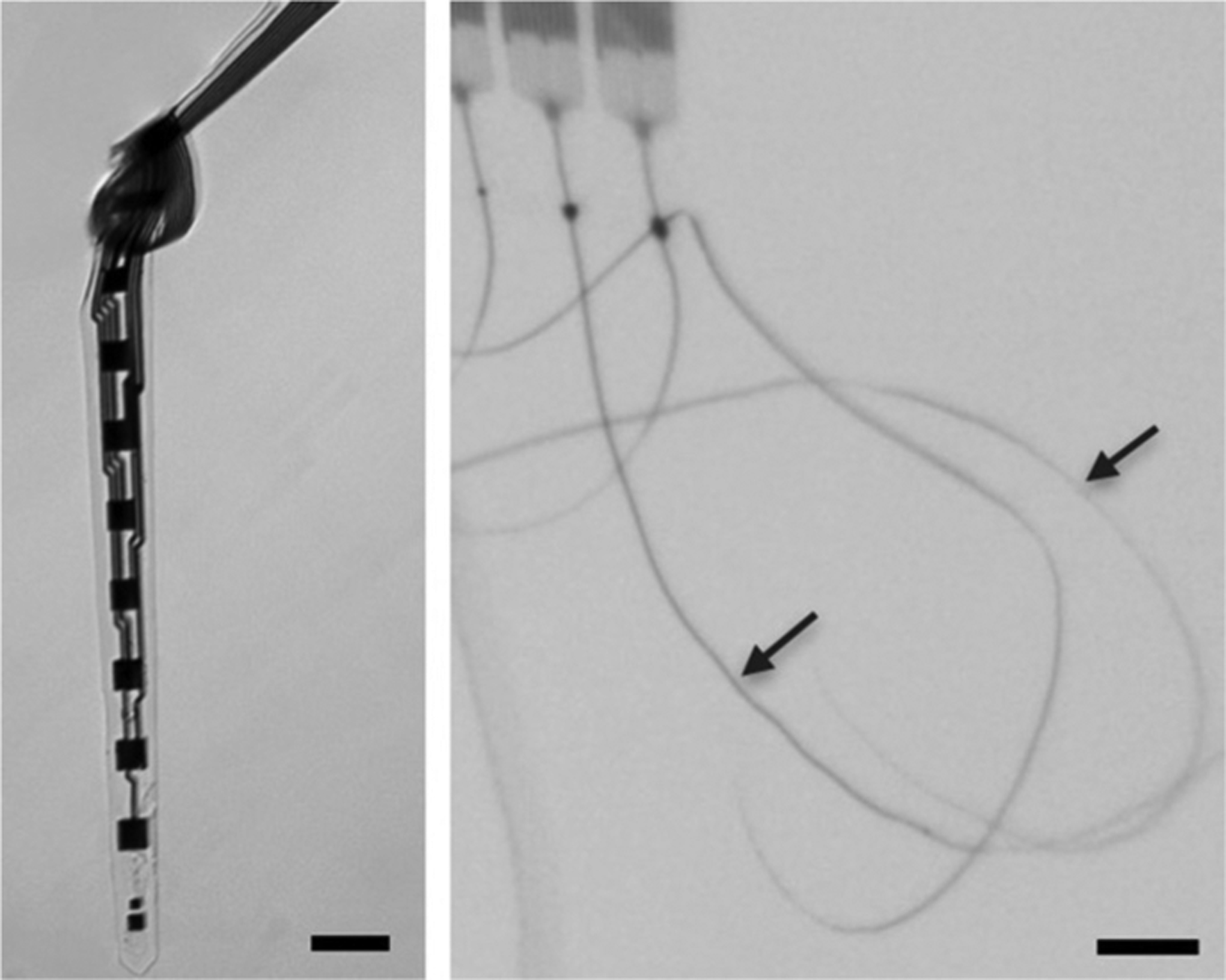
Penetrating polymer-based ultra-flexible electrodes can match the mechanical properties of the brain by virtue of their thin substrates. These types of ultra-flexible probes can minimize the body’s foreign body response and support stable long-term electrical interfaces. (scale bars 10 μm). [65]
